# Inhibiting IL-6 in COVID-19: we are not sure

**DOI:** 10.1186/s13054-020-03177-x

**Published:** 2020-07-27

**Authors:** Patrick M. Honore, Leonel Barreto Gutierrez, Luc Kugener, Sebastien Redant, Rachid Attou, Andrea Gallerani, David De Bels

**Affiliations:** 1 ICU Department, Centre Hospitalier Universitaire Brugmann-Brugmann University Hospital, Place Van Gehuchtenplein, 4, 1020 Brussels, Belgium; 2 Centre Hospitalier Universitaire Brugmann, Brussels, Belgium

We read with great interest the article by Convertino et al. who concluded that the cytokine storm, and especially interleukin-6 (IL-6), is a potentially promising target for pharmacological immunomodulation therapy in COVID-19 acute respiratory distress syndrome (ARDS) [1]. We would like to make some comments, as we believe that different pharmacological immunomodulation approaches are not equal. Indeed, in some patients, disease progression leads to an enormous secretion of cytokines, known as the cytokine storm, and among those cytokines, IL-6 plays an important role [2]. It is important to note that IL-6 has both pro- and anti-inflammatory properties, depending on the pathway of transduction: the classic signaling pathway (mediated by the membrane-bound form of the IL-6 receptor) is believed to be anti-inflammatory, while the trans-signaling pathway (mediated by the soluble form of the IL-6 receptor) is believed to be pro-inflammatory [2]. Given that high levels of IL-6 are associated with lung lesions in COVID-19 patients in the acute and later stages [2], a valid therapeutic option could be to target the expression of IL-6 with tocilizumab, a monoclonal antibody against the IL-6 receptor [2]. However, tocilizumab inhibits both the pro- and anti-inflammatory pathways, and a better option may be to only target the pro-inflammatory (trans-signaling) pathway [2]. Another option is to counteract IL-6 via inhibition of its intracellular transduction pathway with Janus kinase (JAK) inhibitors such as ruxolitinib and baricitinib; however, in doing so the benefit of specifically targeting the pro-inflammatory pathway is again lost [2]. Soluble gp130Fc protein (sgp130Fc), a fusion protein of the gp130 receptor with the Fc portion of a human immunoglobulin antibody, selectively inhibits the trans-signaling pathway [2]. Therefore, sgp130Fc may hold promise as a potential inhibitor of IL-6 in COVID-19 patients, as it could preserve the regenerative and anti-inflammatory properties of the IL-6 classic pathway and block only the pro-inflammatory actions mediated by trans-signaling pathway [3]. It has been shown that blockade of IL-6 trans-signaling using sgp130Fc was as efficient as global blockade of IL-6 classic and trans-signaling using >specific monoclonal antibodies [4]. Inhibition of only the pro-inflammatory pathway of IL-6 seems to be a more reasonable choice than inhibiting both pathways.

## Authors’ response

### Inhibiting IL-6 in COVID-19: we are not sure that sgp130(fc) can affect only the trans-signaling receptor pathway of IL-6

Irma Convertino^1^, Marco Tuccori^1,2^, Corrado Blandizzi^1,2^

1. Unit of Pharmacology and Pharmacovigilance, Department of Clinical and Experimental Medicine, University of Pisa, Pisa, Italy

2. Unit of Adverse Drug Reactions Monitoring, University Hospital of Pisa, Pisa, Italy

We appreciate the letter by Honore et al. and agree that a selective pharmacological approach is more rational than unspecific/non-selective medications. With regard to interleukin-6 (IL-6) targeting, we believe that current research, including the sgp130(fc) development, is rightly going towards this direction. Nevertheless, we wish to point out some considerations.

Firstly, owing to the need of identifying promptly approaches for managing the COVID-19-related acute respiratory distress syndrome (ARDS), our article focused on drugs currently approved for other therapeutic indications, where they target cytokines involved in the COVID-19 inflammatory storm [1].

Secondly, we are well aware that IL-6 acts through both transmembrane IL-6 receptors (tmIL-6R; mainly regenerative or anti-inflammatory functions) and soluble IL-6 receptors (sIL-6R; trans-signaling pathway with pro-inflammatory actions). However, this anti-inflammatory/pro-inflammatory balance of the IL-6 receptor axis can be affected by several variables, such as proteases, other cytokines, concomitant drugs, genetic factors (up/down-regulation of tmIL-6R, sIL-6R, and gp130 expression), with relevant consequences on IL-6 signaling [5]. In this context, a specific/personalized therapeutic approach could be a good option, but it might be more suitable for chronic than acute conditions, such as the COVID-19-related ARDS. Moreover, even though IL-6 trans-signaling can be inhibited selectively, some detrimental conditions resulting from IL-6 blockade, such as serum increments of liver transaminases, lipids, cholesterol, and C-reactive protein, may still occur [6].

Thirdly, current evidence on the benefit of blocking the IL-6 pathway in COVID-19-related ARDS is encouraging. For instance, in a case series, 100 COVID-19 patients with ARDS displayed a prompt improvement after tocilizumab administration, in the setting of both intensive care (74%) and non-intensive care (65%) [7]. We agree with Honore et al. that sgp130(fc) could be a promising therapeutic approach. However, this novel biological drug exerts its inhibiting action on the IL-6 trans-signaling in a dose-dependent manner, and in an in vivo model, when administered at high dosage, it was shown to interfere also with the classical signaling pathway of tmIL-6R [8]. Thus, additional studies are required to identify the most appropriate anti-inflammatory dose of sgp130(fc) that could allow to achieve optimal benefits in the management of hyperinflammation associated with COVID-19-related ARDS.

In conclusion, adequate clinical trials are required to assess whether a more favorable risk/benefit profile is associated with the inhibition of IL-6 trans-signaling, as compared with the overall blockade of IL-6 receptor pathways. While waiting for such evidence, we must continue to rely on currently available pharmacological approaches.

## Data Availability

Not applicable.

## Abbreviations

ARDS: acute respiratory distress syndrome
IL-6: interleukin-6
sgp130Fc: soluble gp130Fc protein
JAK: Janus kinase
tmIL-6R: Transmembrane IL-6 receptor
sIL-6R: Soluble IL-6 receptor
